# Simultaneous monitoring of cerebral metal accumulation in an experimental model of Wilson’s disease by laser ablation inductively coupled plasma mass spectrometry

**DOI:** 10.1186/1471-2202-15-98

**Published:** 2014-08-20

**Authors:** Sorina Georgiana Boaru, Uta Merle, Ricarda Uerlings, Astrid Zimmermann, Sabine Weiskirchen, Andreas Matusch, Wolfgang Stremmel, Ralf Weiskirchen

**Affiliations:** Institute of Clinical Chemistry and Pathobiochemistry, RWTH Aachen University Hospital Aachen, Pauwelsstr. 30, D-52074 Aachen, Germany; Department of Gastroenterology, Internal Medicine IV, University Clinics of Heidelberg, Heidelberg, Germany; Central Institute of Engineering, Electronic und Analytics (ZEA-3), Research Centre Jülich (FZJ), Jülich, Germany; Institute of Neuroscience and Medicine (INM-2), Research Centre Jülich (FZJ), Jülich, Germany

**Keywords:** Wilson’s disease, Bio-imaging, LA-ICP-MS, Copper, ATPase7B

## Abstract

**Background:**

Neuropsychiatric affection involving extrapyramidal symptoms is a frequent component of Wilson’s disease (WD). WD is caused by a genetic defect of the copper (Cu) efflux pump ATPase7B. Mouse strains with natural or engineered transgenic defects of the *Atp7b* gene have served as model of WD. These show a gradual accumulation and concentration of Cu in liver, kidneys, and brain. However, still little is known about the regional distribution of Cu inside the brain, its influence on other metals and subsequent pathophysiological mechanisms. We have applied laser ablation inductively coupled plasma mass spectrometry and performed comparative metal bio-imaging in brain sections of wild type and *Atp7b* null mice in the age range of 11–24 months. Messenger RNA and protein expression of a panel of inflammatory markers were assessed using RT-PCR and Western blots of brain homogenates.

**Results:**

We could confirm Cu accumulation in brain parenchyma by a factor of two in WD (5.5 μg g^−1^ in the cortex) vs. controls (2.7 μg g^−1^) that was already fully established at 11 months. In the periventricular regions (PVR) known as structures of prominent Cu content, Cu was reduced in turn by a factor of 3. This corroborates the view of the PVR as efflux compartments with active transport of Cu into the cerebrospinal fluid. Furthermore, the gradient of Cu increasing downstream the PVR was relieved. Otherwise the architecture of Cu distribution was essentially maintained. Zinc (Zn) was increased by up to 40% especially in regions of high Cu but not in typical Zn accumulator regions, a side effect due to the fact that Zn is to some degree a substrate of Cu-ATPases. The concentrations of iron (Fe) and manganese (Mn) were constant throughout all regions assessed. Inflammatory markers TNF-α, TIMP-1 and the capillary proliferation marker α-SMA were increased by a factor of 2–3 in WD.

**Conclusions:**

This study confirmed stable cerebral Cu accumulation in parenchyma and discovered reduced Cu in cerebrospinal fluid in *Atp7b* null mice underlining the diagnostic value of micro-local analytical techniques.

**Electronic supplementary material:**

The online version of this article (doi:10.1186/1471-2202-15-98) contains supplementary material, which is available to authorized users.

## Background

Wilson’s disease (WD) is a disorder of copper (Cu) disposition of autosomal recessive inheritance [1]. As a consequence, excessive Cu accumulates in tissues and causes organ damage and malfunction which is responsible for the incidence of a wide spectrum of hepatic and neuropsychiatric symptoms. The affected gene, i.e. *Atp7b* is located on the long arm of human chromosome 13 and encodes a Cu-transporting P-type adenosine triphosphatase, ATPase7B, which is beneath ATPase7A the second known Cu efflux pump. In contrast to almost all other nutrients, Cu passively enters the cell via channels such as CTR1 and excess Cu is actively sequestered. In polarized epithelial cell types forming inner surfaces such as gut enterocytes, choroid plexus ependymocytes and also capillary endothelial cells ATPase7A is localized to the basolateral membrane and ATPase7B is localized to the apical membrane [2]. While those contain both isoforms, hepatocytes almost exclusively contain ATPase7B. Consequently, ATPase7A is responsible for absorption of Cu from the intestine into the blood or from the capillary lumen into the tissue parenchyma while ATPase7B is responsible for sequestration of Cu into the bile, into the cerebrospinal fluid or back from an organ parenchyma into the circulation. Cu efflux may sequentially occur by separate sequestration of Cu and Coeruloplasmin apoprotein into trans-Golgi vesicles, followed by formation of the tight complex there and subsequent exocytosis. Although Cu is an essential cofactor for a number of enzymes and required for numerous cellular processes, Cu overload is highly toxic resulting in oxidative damage and inflammation. Within the brain those WD patients with neurological symptoms showed lesions or atrophia in the basal ganglia and other regions [3]. To our knowledge no data is available on Cu concentrations in regions of post mortem human WD brain. The initial neurological symptoms of Cu overload may be very subtle, such as mild tremor, as well as speech and writing problems [4]. At the molecular level these alterations are mainly caused by metal dependent production of dangerous radical oxygen species and the ability of Cu to affect the secretion of molecules involved in the protection of neurons against oxidative stress [5].

There are several rodent models of Cu overload available that generate defects that closely resemble those found in patients suffering from WD. Historically, the first model described were Long-Evans cinnamon rats that carry a transcriptional deficient *Atp7b* mutant in which a deletion removes over 900 bp of the coding region at the 3′ end that encodes the crucial highly conserved ATP binding domain [6–8]. Similarly, the toxic milk mutation in mice in which a single base difference within the *Atp7b* gene causes hepatic accumulation of Cu that can be 100-fold higher than that observed in normal adults is also a well-accepted animal model for WD [9–11]. The respective phenotype of these mice resembles in part that seen in patients with WD [12]. A recent report has shown that the toxic milk mice accumulate Cu in different regions of the brain resulting in an approximately twofold higher mean brain Cu concentration at an age of 12 months than that observed in age-matched control mice [13]. In addition, the same study reported that the cerebral zinc (Zn) concentration in 10 months animals was slightly higher than in control mice and that further iron (Fe) accumulation was found in the hippocampus of older animals. In this model, elevated concentrations of Cu were mainly found in the striatum, hippocampus and cerebellum but not in the cerebral cortex [14]. These alterations were accompanied by a mild impairment in the rotarod and cylinder tests and further by a lack of acquisition of spatial memory in the Morris water maze [14]. In this regard, the phenotype of toxic milk mice resembles the clinical symptoms of patients suffering from WD. However, this model has some limitations. First, brain lesions in 12-month-old mice are not as advanced as in patients with the neurological form of WD [13]. Second, pups are born Cu-deficient and the milk produced by mutant mothers contains too low amounts of Cu resulting in early death, a characteristic that is not seen in patients affected by WD [9]. Moreover, morphologic and chemical studies of hepatic Cu toxicosis indicated striking differences in morphologic integrity between regenerative nodules and the intervening parenchyma with profound changes in mitochondria, endoplasmic reticula, and nuclei as well as the accumulation of microvesicular lipid droplets in injured hepatocytes, again features that are not seen in patients with WD [15]. In addition, despite a significant elevation of Cu in the brain of the Cu-loaded toxic milk mice no behavioral changes were observed in other studies [10] raising some doubts about whether this model is appropriately mimicking WD [15].

*Atp7b* deficient mice that were generated by directed genetic disruption display a gradual accumulation of hepatic Cu that increases to a level 60-fold greater than normal by 5 months of age and develop cirrhosis of the liver, while the progeny of homozygous mutant females are initially characterized by neurological abnormalities and growth retardation that is specific to Cu deficiency [16]. In addition, it is known that these mice show an increase in Cu and Fe concentration in the brain [16]. Interestingly, these mice have further elevated hepatic and cerebral superoxide dismutase activities indicating oxidative stress in respective tissues that is combined with a severe dysfunction of mitochondrial energy production [17].

Using quantitative positron emission tomography in *Atp7b* deficient mice, it was demonstrated that injection of ^64^CuCl_2_ intravenously resulted in increased accumulation and markedly reduced clearance of Cu from the liver, while ^64^Cu radioactivity at all times was lower in the brain, kidneys, lungs, and heart compared to wild type (WT) animals suggesting that the reduced ^64^Cu radioactivity in the *Atp7b* null mice in extrahepatic tissues may be caused by hepatic trapping of Cu [18]. Likewise, the oral administration of radioactive CuCl_2_ was not able to induce higher concentrations of Cu within brains of mice that lack functional ATP7B protein [19]. These imaging findings demonstrate that Cu homeostasis and distribution in different organs is differentially regulated.

Laser ablation inductively coupled plasma mass spectrometry (LA-ICP-MS) is a method capable of simultaneously detecting numerous metals and non-metals in one sample at concentrations as low 0.1–1 μg g^−1^ for Mn, Fe, Cu, Zn and 0.05–0.2 μg g^−1^ for lanthanides [20, 21].

In the present study we performed LA-ICP-MS measurements in brains isolated from *Atp7b* deficient mice and respective controls. Furthermore, gene expression and protein levels of a panel of inflammatory and blood vessel proliferation markers were studied.

## Results and discussion

Metal accumulation in the brain and liver is a well known consequence of WD. Studies using magnetic resonance imaging have shown that patients with WD show bilateral T2 hyperintensity involving thalami, cerebral white matter, basal ganglia, cerebellum and brainstem [22]. Cu loading studies in the toxic milk mouse model (a natural defect of ATPase7B) have shown that there are some chronological changes in tissue Cu loading that also affect the bio-distribution of other metals [10]. A recent publication analyzing this model has further shown that the amount of Cu overload was unequally distributed in different brain regions [14]. While the concentration was increased in the striatum, hippocampus and cerebellum, the concentration of Cu was unaltered in the cerebral cortex [14]. However, all these former studies used atomic absorption spectroscopy to measure metal concentrations in total extracts of the brain or its different areas and a more precise study of Cu distribution in brain and its impact on the cerebral concentration of other metals or metalloids is presently not available in any experimental models of WD.

**Figure 1:**
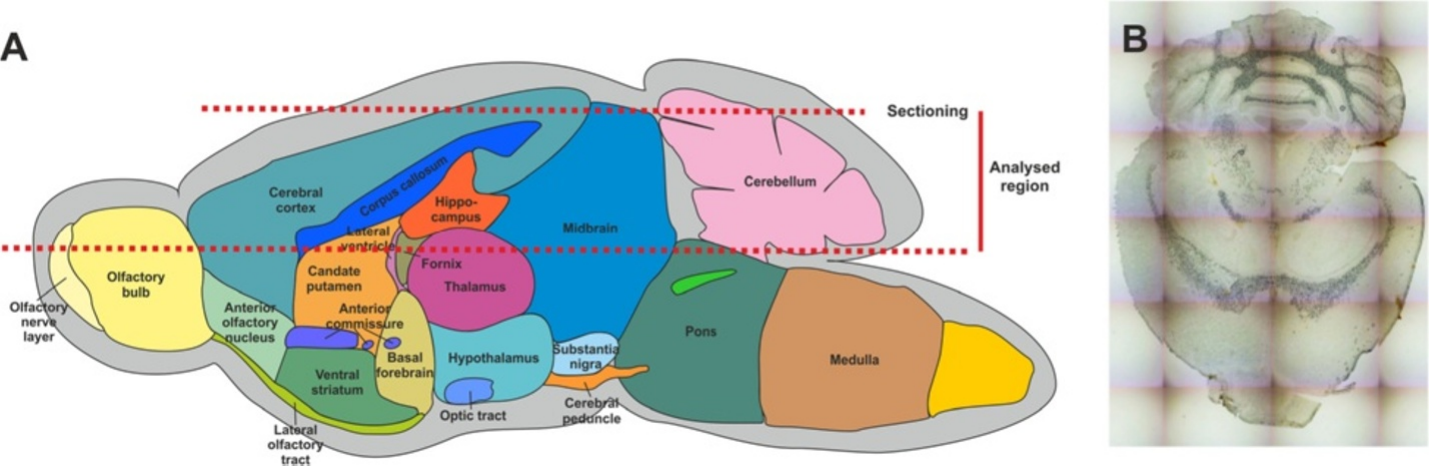
**Schematic overview of the murine brain and position of the sectional plane used for preparing cryosections. (A)** In the scheme, the different regions and the architecture of the mouse brain are depicted. The red lines mark the position for sectioning and the region from which horizontal cryosections were prepared. **(B)** A photomicrograph of a representative cryosection used for imaging is shown.

Recently, we have shown that metal bio-imaging via LA-ICP-MS is a novel, highly sensitive innovative diagnostic tool that allows simultaneous measurement of over fifty different metal concentrations in liver tissue with a high sensitivity, spatial resolution, specificity, and quantification ability [23, 24]. We here used this methodology to get more insight into the cerebral distribution of various metals during progression of WD. To do so, we comparatively imaged brain sections of WT and *Atp7b* deficient mice at different ages by LA-ICP-MS. The horizontal sections analyzed contained cortex, corpus callosum, hippocampus, colliculus superior, cerebellum and all four ventricules in all cases, colliculus inferior in most cases, striatum, thalamus and brain stem structures in some cases (Figure 1).

Since numerous findings have already uniformly suggested that neuropathological alterations such as found in Alzheimer’s disease, Down syndrome, Parkinson’s disease, multiple sclerosis, and many other neurodegenerative disorders are characterized or even causatively driven by altered expression of IL-1β [25–27], we first comparatively tested the mRNA expression of this cytokine in WT and *Atp7b* deficient mice (Additional file 1: Figure S1A). In contrast to all these previous reports, we found that the IL-1β mRNA quantities were only slightly but not significantly elevated in WD vs. controls. TNF-α mRNA expression, in turn, was significantly increased more than two-fold in WD. TNF-α is associated with damage of myelin, oligodendrocytes and astrocytes [28, 29] and may possibly reflect ongoing neurotoxicity [30]. In line with this hypothesis, we found increased expression of the NLRP3 and ASC inflammasome components in the brains of WD mice. Those components have important roles in the pathology of microglia-specific formation of neurodegenerative disorders and further regulate CNS inflammation and de-myelination during cerebral insult [31, 32]. This finding again demonstrates that affected mice have exacerbating CNS inflammatory activity within their brains.

In addition, both, the tissue inhibitor of metalloproteinase-1 (TIMP-1) and the matrix metalloproteinase 9 (MMP-9) that represent biomarkers for the presence or extent of brain injury and are necessary for the clearance of amyloid [33, 34] were expressed at higher levels in *Atp7b* deficient brains. Since MMPs promote blood–brain barrier leakage, it is possible that the elevated expression of MMP-9 may already indicate existing brain cell dysfunction and cerebral cell death [35].

We next performed Western blot analysis and found that the expression of α-smooth muscle actin (α-SMA) in protein extracts prepared from the brains of *Atp7b* knockout mice (cf. Figure 1) was significantly higher (Additional file 1: Figure S1B). In normal brain tissue, α-SMA is generally undetectable and it was previously supposed that α-SMA should be considered as a marker for smooth muscle cell differentiation, which in the brain becomes detectable in areas with microvascular proliferation [36]. Again, these findings indicate that the *Atp7b* nulls already show signs of cerebral dysfunction. The increased activation of inflammatory pathways within the brains that we have already observed at the transcriptional level was also noticeable in the elevated concentration of the lipocalin 2 (LCN2) in older animals that lack *Atp7b*. We have recently demonstrated that the upregulation of LCN2 is a reliable indicator of organ damage that is significantly correlated to inflammation [37, 38]. Altogether, our gene and protein expression data indicate that the *Atp7b* deficient mice have some cerebral alterations that are characteristic for WD in humans.

**Figure 2:**
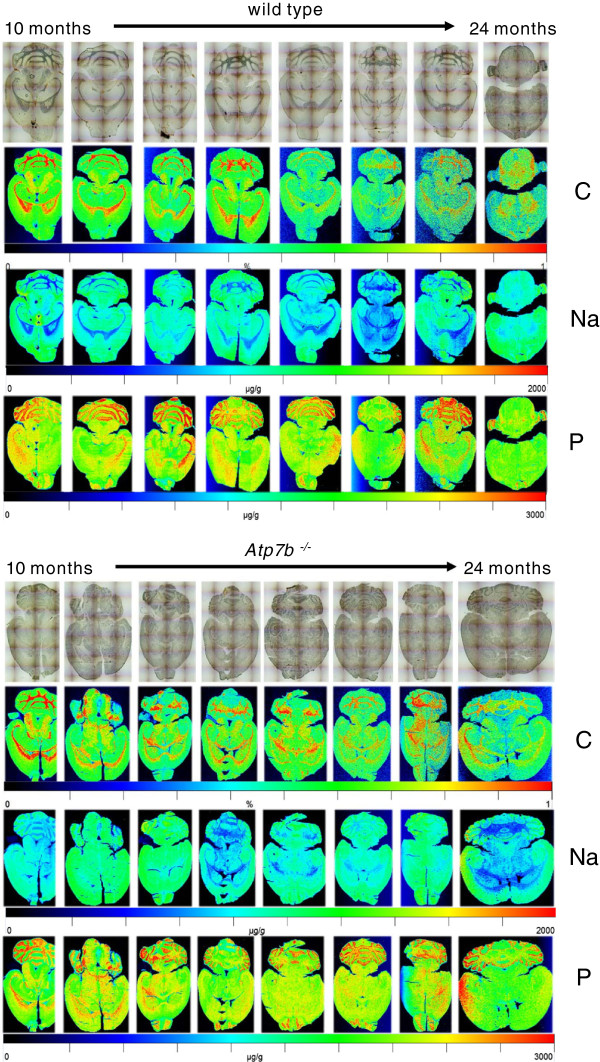
**Bioimaging of sodium and phosphate in the murine brain.** Thin brain section of WT (*upper panel*) and *Atp7b* deficient mice (*lower panel*) were prepared by cryo-cutting and analyzed for Na and P content by LA-ICP-MS. In these measurements, the metal intensities were normalized to the average ^13^C ion intensity in respective samples. The photomicrographs of the analyzed cryo-sections are depicted for orientation.

**Figure 3:**
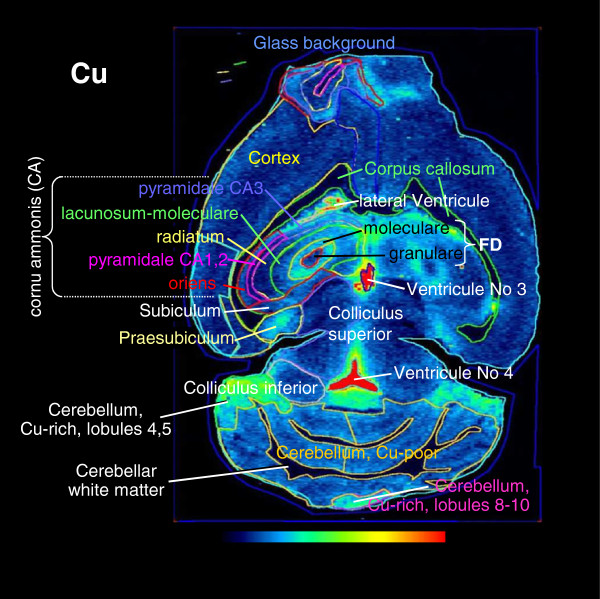
**Regions of interest.** Exemplarily different regions of the murine brain in which metal content was measured by LA-ICP-MS and quantified (see also Tables 1 and 2) are depicted.

**Figure 4:**
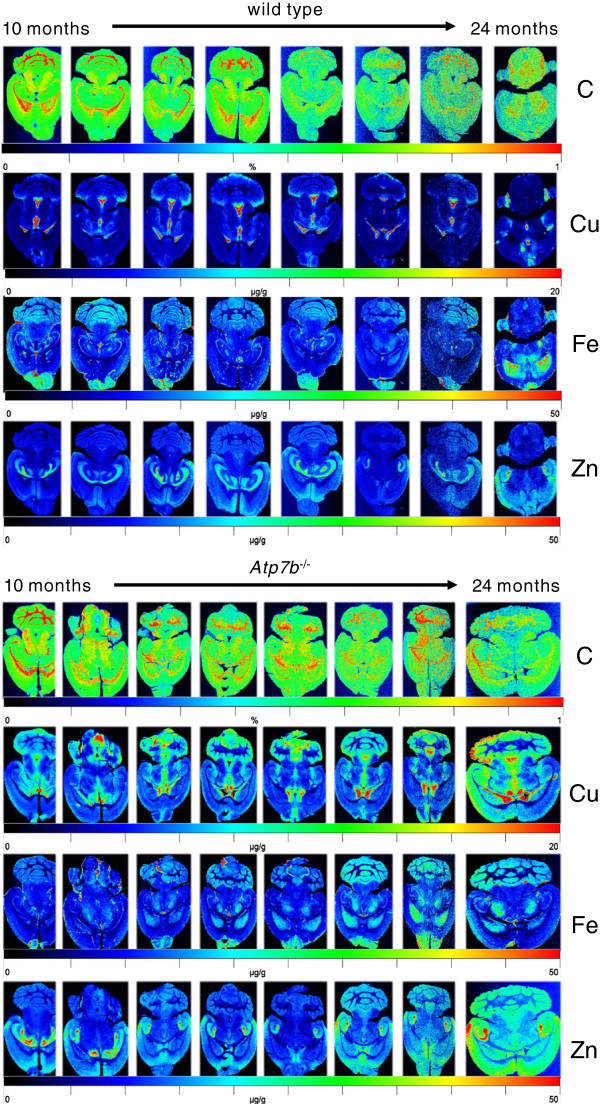
**Age-dependent cerebral accumulation of copper in** ***Atp7b*** ^**−/−**^ **mice as demonstrated by LA-ICP-MS.** 30 μm thick cryosections from WT (*upper panel*) and *Atp7b* ^−/−^ mice (*lower panel*) at age between 10 and 24 months were subjected to LA-ICP-MS imaging. The content of carbon that served as reference in this analysis is given in %, while the total concentrations of Cu, Zn, and Fe are given in μg g^−1^ tissue.

**Figure 5:**
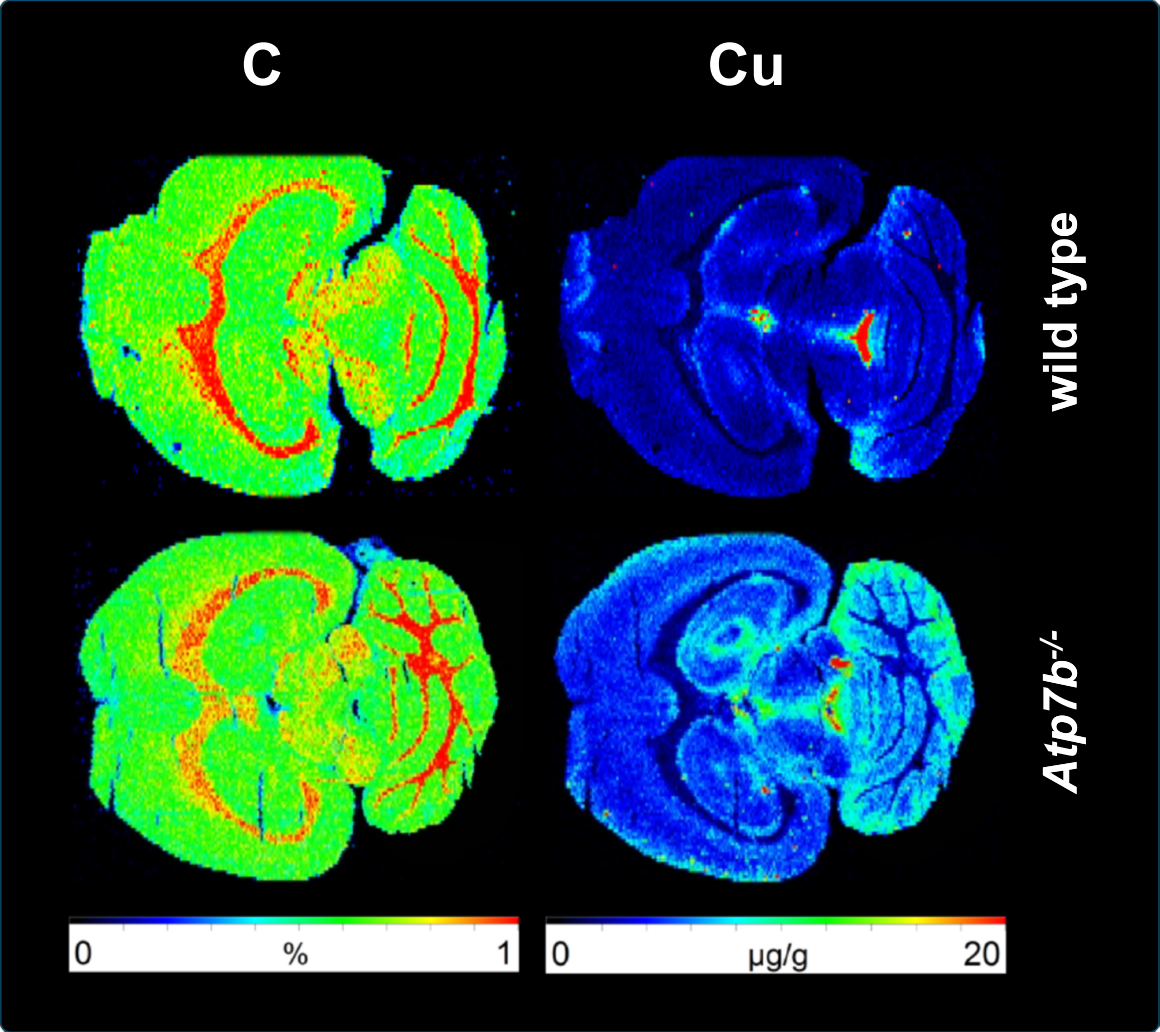
**High resolution imaging of copper within the brain of age-matched wild type and** ***Atp7b*** **deficient mice.** Brain sections of a WT (*upper panel*) and *Atp7b* deficient mice (*lower panel*) were prepared from age-matched (~11 months) old animals and subjected to LA-ICP-MS imaging to estimate the content of copper. Carbon served as an internal standard in this analysis.

LA-ICP-MS simultaneously yielded images of the 28 preselected m/z. Amongst these elements we first inspected the distribution of sodium and phosphorus within the brains of WT and *Atp7b* deficient mice in cryosections with a thickness of 30 μm (Figure 2) [39] resulting in a maintained architecture of these elements. In the unchanged physiological situation highest concentrations of phosphorus (up to 3,000 μg g^−1^) were found in the granular layer of the cerebellum forming the shape of the arbor vitae, the granular layer of the olfactory bulb, in the intrastriatal and intracollicular fiber bundles and corpus callosum (Figure 2). Sodium in the well known manner showed the distribution of water with higher concentrations in grey matter 800 μg g^−1^ compared to white matter 600 μg g^−1^. Inversely, ^13^C ion intensities reflected the distribution of dry organic matter congruent with the distribution of the fatty white matter. Next manganese (Mn), Fe, Cu and Zn were assessed quantitatively in the regions of interest exemplarily depicted in Figure 3. Average concentrations read out from large regions are given in Table 1 and those of small hippocampal regions in Table 2. While Fe and Mn were unchanged and of perfectly preserved distributional architecture a tremendous increase of Cu was already obvious from visual inspection (Figures 4 and 5). At a second glance the decrease of Cu in the periventricular regions was obvious, especially in the fourth ventricule where a lumen was systematically discernable in WD animals but not in controls. Otherwise the physiological distribution pattern of Cu was maintained. This metal is the element presenting the highest range of physiological concentrations. In WT controls Cu ranged from a minimum in callosal white matter of 1.5 μg g^−1^ to 67 μg g^−1^ in the fourth ventricule. In the other grey matter components Cu occurs in quite low concentrations such as 2.7 μg g^−1^ in the cortex and 3.3 μg g^−1^ in the colliculus superior. Somewhat higher Cu was observed in the stratum moleculare of the fascia dentata (5.2 μg g^−1^), the preasubiculum (4.8 μg g^−1^) and in the glomerular layer of the olfactory bulb. Within the isocortex Cu was slightly higher in the layers 2, 3 and 6. For validation additional 3 WD mice vs. 3 age-matched controls were studied by the generation of quantitative images only but not by region of interest based analysis. Here especially high quality Mn images were obtained and all above mentioned findings could be reproduced (Additional file 2: Figure S2).

**Table 1:** **Average concentration of Fe, Cu, Zn and Mn in large regions of the brain**

μg g^−1^wet weight	Control	WD	%** change	*p*- value
Entire section				
Fe	14.1 ± 2.5	14.1 ± 1.0		.9
Cu	4.2 ± 1.4	6.7 ± 1.5	+61%	.0007
Zn	13.2 ± 3.1	15.8 ± 1.7	+19%	.07
Mn	0.25 ± 0.07	0.26 ± 0.05		.4
Cortex (grey matter)				
Fe	12.4 ± 2.2	12.1 ± 0.8		.7
Cu	2.7 ± 0.7	5.5 ± 1.2	+108%	.00004
Zn	13.9 ± 3.0	17.7 ± 0.8	+28%	.008
Mn	0.23 ± 0.06	0.26 ± 0.04		.2
Corpus callosum (white matter)*				
Fe	7.8 ± 1.1	7.6 ± 0.6		.6
Cu	1.5 ± 0.5	2.9 ± 0.8	+102%	.0004
Zn	8.2 ± 2.1	9.5 ± 1.4		.2
Mn	0.11 ± 0.03	0.13 ± 0.04		.2
Cerebellum, Cu rich regions lobules 4–5, 8-10				
Fe	17.4 ± 3.8	17.4 ± 2.1		1
Cu	6.6 ± 1.6	10.9 ± 1.7	+64%	.0001
Zn	13.0 ± 2.9	16.0 ± 2.0	+23%	.03
Mn	0.28 ± 0.09	0.28 ± 0.04		.9
Cerebellum Cu poor regions (other)				
Fe	17.8 ± 3.5	16.3 ± 3.8		
Cu	3.8 ± 0.9	7.5 ± 1.6	+95%	.0002
Zn	12.3 ± 2.7	13.8 ± 3.1		.3
Mn	0.30 ± 0.08	0.27 ± 0.06		1
Cerebellar white matter*				
Fe	8.4 ± 1.9	6.9 ± 1.9		.1
Cu	1.7 ± 0.3	4.0 ± 1.2	+132%	.0004
Zn	6.8 ± 1.4	7.9 ± 1.2		.1
Mn	0.15 ± 0.04	0.13 ± 0.06		.9
Colliculus superior				
Fe	13.8 ± 2.2	13.3 ± 1.1		.7
Cu	3.3 ± 0.7	6.1 ± 0.5	+85%	.0005
Zn	9.8 ± 2.0	11.4 ± 0.4		.08
Mn	0.31 ± 0.07	0.34 ± 0.07		.3
Colliculus inferior				
Fe	15.7 ± 3.4	12.1 ± 1.5		.05
Cu	3.6 ± 0.9	5.4 ± 0.7	+49%	.003
Zn	9.5 ± 2.1	11.2 ± 1.7		.1
Mn	0.36 ± 0.10	0.35 ± 0.09		.06
Periventricular regions				
Lateral ventricules				
Cu	39 ± 17	17 ± 3	−56%	.01
Third ventricule				
Cu	50 ± 28	22 ± 4	−57%	.02
Fourth ventricule				
Cu	67 ± 22	19 ± 5	−71%	.0004

Concentrations of Fe, Cu, Zn, Mn (mean ± SD) measured by imaging LA-ICP-MS in regions of interest in the brain of 8 controls, and 9 WD mice, both on a SVJ129 background. Of each animal, one section was measured whereby each hemisphere was measured in an independent run with separate calibration standard. SD and heteroskedastic t-tests were calculated over the 8 and 9 individual bi-hemispheric averages.

*white matter borders were delineated to minimize spillover from neighbour regions.

**indicated, if > +10% or < −10% and p < .05.

**Table 2:** **Average concentration of Fe, Cu, Zn and Mn in small hippocampal regions of the brain**

μg g^−1^wet weight	Control	WD	% change	p- value
Fascia dentata, layers				
Moleculare				
Fe	13.1 ± 2.1	14.6 ± 1.2		.1
Cu	5.2 ± 1.2	8.9 ± 1.2	+71%	.00001
Zn	14.0 ± 3.7	19.4 ± 1.3	+38%	.004
Mn	0.34 ± 0.10	0.32 ± 0.07		.9
Granulare				
Fe	18.4 ± 3.4	16.3 ± 2.1		.2
Cu	4.1 ± 1.1	7.6 ± 1.0	+86%	.00001
Zn	25 ± 7	32 ± 3	+30%	.02
Mn	0.39 ± 0.14	0.39 ± 0.09		.7
Multiforme /polymorph				
Fe	12.9 ± 1.7	13.2 ± 0.8		.7
Cu	4.1 ± 1.2	8.0 ± 1.5	+95%	.0001
Zn	47 ± 14	59 ± 9	+24%	.1
Mn	0.48 ± 0.28	0.48 ± 0.14		.8
Cornu ammonis, layers				
Oriens				
Fe	10.8 ± 1.2	10.6 ± 1.0		.7
Cu	3.8 ± 0.8	6.6 ± 1.4	+75%	.0002
Zn	18.0 ± 4.0	20.8 ± 3.8		.2
Mn	0.26 ± 0.06	.32 ± 0.08		.04
Pyramidale, CA3				
Fe	11.3 ± 1.9	12.3 ± 1.4		.3
Cu	4.6 ± 1.4	7.0 ± 1.2	+52%	.003
Zn	33 ± 10	38 ± 5.5		.2
Mn	0.45 ± 0.20	0.48 ± 0.13		.5
Pyramidale, CA1-2				
Fe	19.0 ± 3.7	15.1 ± 1.5		.03
Cu	3.5 ± 1.0	5.9 ± 0.9	+69%	.0002
Zn	20.9 ± 4.8	24.4 ± 3.4		.1
Mn	0.27 ± 0.07	0.33 ± 0.06		.04
Radiatum				
Fe	10.5 ± 1.5	10.2 ± 0.7		.7
Cu	3.2 ± 0.8	6.3 ± 1.2	+92%	.00002
Zn	21.8 ± 4.9	23.8 ± 3.0		.3
Mn	0.25 ± 0.06	0.31 ± 0.08		.04
Lacunosum- moleculare				
Fe	12.8 ± 2.1	12.2 ± 1.3		.6
Cu	3.6 ± 0.9	5.6 ± 1.0	+53%	.001
Zn	14.1 ± 3.7	18.4 ± 1.5	+30%	.01
Mn	0.28 ± 0.08	0.29 ± 0.06		.4
Subiculum				
Fe	14.4 ± 2.3	12.9 ± 1.4		.2
Cu	3.4 ± 0.9	5.9 ± 1.1	+72%	.0001
Zn	15.4 ± 4.1	17.4 ± 1.6		.2
Mn	0.23 ± 0.06	0.27 ± 0.03		.7
Praesubiculum				
Fe	13.9 ± 3.2	13.8 ± 1.5		1
Cu	4.8 ± 1.4	7.8 ± 1.4	+62%	.0006
Zn	13.1 ± 3.0	16.3 ± 1.0	+25%	.02
Mn	0.23 ± 0.07	0.26 ± 0.05		.2

Concentrations of Fe, Cu, Zn, Mn (mean ± SD) measured by LA-ICP-MSI in subregions of the hippocampal formation. Layers are given in the order from outside to inside.

Quantitative analysis confirmed an almost proportional increase of Cu by 50%-130% in the brain parenchyma of *Atp7b* deficient animals without further obvious systematic between more or less Cu rich regions. This finding reached significances of *p* = 0.00004 fulfilling even the most conservative Bonferroni criteria. In the periventricular regions in turn Cu was decreased by factors of 2.3 in and around ventricules I-III and of 3.5 around ventricule IV. Interestingly, also the gradient of Cu concentrations increasing downstream from lateral over third to fourth ventricule was relieved. In the choroid plexus the cerebrospinal fluid (CSF) is produced essentially by ultrafiltration through fenestrated capillaries. A part of the initially co-filtrated Cu is immediately re-absorbed through ependymocytes which express high amounts of ATPase7A at their basolateral membrane. Ependymocytes of the ventricular walls sequester Cu from brain parenchyma to the CSF via ATPase7B localized at their apical portion. It is fully compatible with the view of periventricular regions as Cu efflux compartments that a lack of function of ATPase7B leads to a decrease of Cu there.

Zn was increased by up to 40% especially in regions of high Cu such as the stratum moleculare of the fascia dentata or Cu rich areas of the cerebellum but not in typical Zn accumulator regions, such as the stratum multiforme of the fascia dentata and the CA3 segment of the pyramidal layer of the cornu ammonis, a side effect due to Zn being to some degree a substrate of Cu-ATPases. On the other hand a significant increase of Zn was observed in the Zn rich ectorhinal cortex (from18.8 in WT to 25.6 μg g-1 in WD, p = 0.008) and anterior cingulate cortex (from 18.3 in WT to 23.2 μg g-1 in WD, p = 0.009).

To avoid potential misinterpretation of results that may occur from animal-to-animal variations, we further performed confirmatory measurements in additional 3 WD and 3 WT controls that were at age of 19–24 months. The visual inspection of the images that were generated by the in house written Excel algorithm for visualization of metal concentrations (see Methods) results in similar images that confirmed our measurements (Additional file 3: Figure S3 and data not shown). Therefore, these measurements were not further submitted to region of interest based analysis.

When it comes to variation of metal concentrations with age, the number of sections and animals submitted to quantitative data analysis here is too small for final conclusions. However, it can already be stated that in this sample the full extent of altered Cu disposition was already reached at 11 months and concentrations of Cu, Zn and Mn rather slightly fall with age while Fe was constant in WD. The more interesting period from 0 to 11 months was not covered by this sample. Here, in a previous study in which selected elements were quantified by atomic absorption spectroscopy [17] some of us reported a progressive increase of Cu and Zn. The animals used were aged 3, 6, and 47 weeks (7 animals per group). Fe had reached its full level already in 6 week old mice [17].

Based on our findings, we could confirm that the homozygous *Atp7b* deficient mice used in our study accumulate cerebral Cu. A previous study that has reported the establishment of the respective animal model has already demonstrated by use of polarized atomic absorption spectrophotometry of dried tissue samples that the levels of Cu in brain of homozygous mutant animals increased slightly throughout adult life from 12.6 ± 6.8 to 36 ± 4 μg g^−1^ dry weight [16]. In human WD [40], the functionality controlled by regions such as the basal ganglia, namely striatum and pallidum is tremendously affected potentially indicating that the observed alterations might be a direct consequence of Cu deposits there [4]. In line with this, the olfactory bulb that is located in the forebrain contains specialized sensory cells and interneurons responsible for accurate transmission of smell information [41]. In WD patients with neurological symptoms a significant irreversible olfactory dysfunction that correlates with Cu content is documented [42]. It was recently demonstrated that increased brain Cu in the toxic milk mice model correlated well with the expression of inflammatory markers and behavioral changes [14]. Immunohistochemical determination of ATP7B protein in brain tissue of rats has shown that this Cu-transporting P-type ATPase was highly expressed in neuronal cells of the hippocampal formation, olfactory bulbs, cerebellum, cerebral cortex and nuclei in the brainstem [43]. The finding that Cu is also enriched in the cerebral cortex when *Atp7b* is absent confirms the hypothesized essential role of this gene in the local export of Cu from cells [44].

Somewhat in contrast to our findings is the observation that intravenous injection of ^64^CuCl_2_ via the tail vein resulted in consistently lower Cu radioactivity at all times in the brain of *Atp7b* knockout mice as compared to control mice when analyzed by quantitative positron emission tomography [19]. In this context, it has to be well discriminated that this PET study measured the uptake and turnover but not the total inventory of Cu as it was the case in our study. Bio-distribution studies with application of radio-Cu at lifetime, victimization after hours and few days and autoradiography of brain sections clearly localized the fastest and highest uptake of Cu to the periventricular regions [45]. Thus lower uptake of Cu into periventricular regions in WD is perfectly in line with the finding of lower Cu there in this study in WD mice.

Another report that was conducted in the toxic milk mouse model on the C3H background has demonstrated that the Cu accumulation was mainly found in striatum, hippocampus and cerebellum, but unaltered in cerebral cortex [14]. The latter finding may be explained by a systematic contamination/mix-up of dissected cortical material with periventricular material (lateral ventricles) and thus wrongly high cortical Cu levels in controls. These mice showed motor and cognitive disturbances most likely because of the Cu deposition and inflammatory response observed in the striatum.

Similarly, accumulation of Fe in the brain of aged LEC rats (also bearing a defect of ATPase7B) was reported previously with highest levels in the striatum and the substantia nigra [46].

There is no doubt that the LA-ICP-MS imaging technology used in this study is transferable to other experimental models, organs, species, and various metal overload diseases. We here used mouse that lacked the plasma membrane Cu-transport protein ATP7B. In future it will be interesting for us to visualize the metal content in supplementary rodent models of WD including the toxic milk mouse model or the Long-Evans cinnamon rats by LA-ICP-MS. In addition, recent reports have provided strong evidence that Cu metabolism Murri domain-containing protein 1 (COMMD1)-deficiency in dogs lead to hepatic Cu accumulation with a WD-like phenotype [47]. Also these models in conjunction with LA-ICP-MS will help to increase the knowledge in the pathogenesis of neurodegenerative disorders that occur during the course of metal accumulation.

## Conclusions

Our study evidenced an about 2-fold stable increase of Cu throughout brain parenchyma in ATPase7B null mice more or less proportional across the regions assessed maintaining the characteristic distribution pattern while in periventricular regions Cu was decreased by down to a factor of 3.5. As Cu was increased proportionally throughout all cerebral regions the regionally and basal ganglia characteristic symptoms of WD may rather result from differential regional susceptibility to Cu than from differential affection of Cu-efflux.

Zn was increased by 10%-40%. It is now tempting to assess horizontal sections at a deeper level covering the candidate structures pallidum, striatum and brain stem nuclei and completing cohort by animals of younger ages. Animal models deficient in Cu transporters and LA-ICP-MS imaging are a setting perfectly suited to study therapeutic interventions for normalizing Cu disposition and also studying their side effects on other metals, namely Zn. In a wider perspective, this study demonstrates the superiority of microlocal analytical techniques over the study of homogenates especially if regions or layers of highly differential analyte concentrations are closely adjacent which cannot be dissected reliably from each other. LA-ICP-MS imaging is an important novel method that will be helpful in the setting of experimental and clinical research. This method will be easily transferable to other models, organs and diseases and will have a strong influence on basic research and diagnostics in the near future.

## Methods

### Animals

The characteristics of *Atp7b* null mutation mice that were used in this study are described elsewhere [16]. In brief multiple stop codons covering all possible reading frames were inserted into exon 2 of the *Atp7b* gene. The transgenic −/− mice express the respective mRNA which is not translated at all into full length functional protein but into considerable smaller non functional proteins. Mutant mice and age-matched controls had a genetic 129/Sv background and were housed at the University of Heidelberg, according to the guidelines of the Institutional Animal Care and Use Committees and in accordance with governmental requirements [17]. For our study, we analyzed a total of 17 animals (controls n = 8; *Atp7b*^−/−^ n = 9) with an age between 11 and 24 months. Confirmatory additional 3 WD mice and 3 controls, aged 19–24 months were analyzed up to the level of quantitative element images but not submitted to region of interest based analysis.

### RNA isolation and quantitative real time PCR

RNA from total brain tissue was isolated by the guanidine thiocyanate/CsCl method, followed by DNAse digestion using the Purelink RNA Mini Kit system (Invitrogen, Life Technologies, Darmstadt, Germany). Total RNA was quantified and 2 μg samples were reversely transcribed using Superscript II reverse transcriptase and random hexamer primers (both from Invitrogen). For the individual TaqMan PCR assays, the cDNA derived from 25 ng RNA was amplified in 25-μl volume using qPCR Core Kits (Eurogentec, Cologne, Germany) and primer combinations given in Additional file 4: Table S1. The amplification of all respective target gene sequences were done as follows: melting at 95°C for 10 min and then 40 cycles at 95°C for 15 sec and 60°C for 1 min, respectively. All samples were normalized to the expression of GAPDH.

### Western blot analysis

Protein extracts from brain tissue were prepared following standard protocols. Equal amounts of proteins (25 μg) were heated at 80°C for 10 min and separated in 4-12% Bis-Tris gels (Invitrogen) under reducing conditions. Proteins were then electro-blotted on nitrocellulose membranes (Schleicher & Schuell, Dassel, Germany). Successful protein transfer and equal protein loading was monitored via Ponceau S stain. Unspecific binding sites were blocked in TBST [10 mM Tris/HCl, 150 mM NaCl, 0.1% (v/v) Tween 20, (pH 7.6)] containing 5% (w/v) non-fat milk powder and the membranes were subsequently probed with the antibodies given in Additional file 5: Table S2. Primary antibodies were detected with horseradish-peroxidase (HRP)-conjugated secondary antibodies (Santa Cruz, Santa Cruz, CA) and the Supersignal chemiluminescent substrate (Perbio Science, Bonn, Germany).

### LA-ICP-MS imaging of trace metals in murine brain sections

In the experimental setup, a quadrupole-based inductively coupled plasma mass spectrometer (ICP-MS, XSeries 2, Thermo Scientific, Bremen, Germany) was coupled to a laser ablation system (NWR 213, New Wave Research, Fremont, CA, USA). For metal imaging, native sections of mouse brains (30 μm thickness) were prepared and thaw mounted onto Starfrost™ adhesive slides. Laser ablation of biological tissue was performed as described before [23] in continuous line ablation mode in pure argon atmosphere at 36% output energy, 60 μm spot size and 30 μm residual between lines making a y-pixel dimension of 90 μm. The ablated material was transported by Ar into the inductively coupled plasma (ICP). Sample gas flow was 1.1 L/min, auxiliary gas flow 0.7 L/min and cooling gas flow 14 L/min. The ions formed in the atmospheric pressure ICP were extracted in the ultrahigh vacuum mass spectrometer via a differential pumped interface, separated in the quadrupole mass analyzer according to their mass-to-charge ratios and detected by an ion detector. The sum of dwell times of 28 m/z for one cycle was 0.341 s thus making an x-pixel dimension of 70 μm s^−1^ × 0.341 s = 24 μm. For quantification purposes, matrix-matched laboratory standards of homogenized brain tissue with well defined element concentrations were prepared as described previously [20, 23, 24, 48]. Also LA-ICP-MS parameters were optimized in an established way [48]. To validate the metal ion images, two isotopes of the same element were simultaneously analyzed when possible. Of each animal, one section was measured whereby each hemisphere was measured in an independent run with a separate calibration standard. Images were reconstructed and calibrated using an in house written Excel algorithm for visualization (Figures 2, 3, 4 and 5, Additional file 2: Figure S2) whereby the average net ^13^C ion intensity, as a surrogate of slice thickness [48], was determined from histograms of pixel values and served for normalizing metal intensities. In an independent approach for validation, raw data were reconstructed into 8 bit grayscale TIFF and, when necessary, linearly drift corrected (the end of the sample next to the standard was kept at a factor of 1 and the correction factor progressively increased or decreased line by line towards the other end) using IMAGENA [49]. The regions of interest including glass background and entire section were defined and average ion intensities read out using Pmod 3.1 (Pmod, Zurich, Switzerland). The observer blinded careful manual delineation of neuroanatomically defined regions of interest used the mutual information from C, P, Mn, Fe, Cu, Zn images, the microphotograph obtained before ablation and the RB4 Watson Paxinos Atlas (download link: http://www.callisto-science.org/NSI/Neuroscience_Image_Database/RUN_RBSC.PDF). Standard measurements were processed by an Excel algorithm yielding slope of the calibration curve. Data were calibrated according to concentration = (maximum counts –minimum counts) × (Ion intensity-background)/(255 × slope). In the present series normalization of sample and standard to the respective net average ^13^C ion intensity produced the same group averages but a higher scatter of individual results. Therefore, non ^13^C normalized data are given as numeric results.

### Statistics

Standard deviations, averages, % change vs. controls and heteroscedastic t-tests for independent samples over the 9 vs. 8 individual bi-hemispheric averages of regional element concentrations were calculated using Excel. The classical Bonferroni threshold for 21 regions × 4 elements was *p* = 0.0006. For the Holmes modified Bonferroni correction the significance threshold for the first region was considered at *p* = 0.05, for the second 0.05/2, for the third 0.05/3 and so on.

## Electronic supplementary material

Additional file 1: Figure S1: Comparative analysis of gene and protein expression in brains of wild type and *Atp7b* ^-/-^ mice. (A) Relative mRNA expression of IL-1β, TNF-α, NLRP3, ASC, TIMP-1 and MMP-9 expression in brain of WT and *Atp7b* deficient mice. Significance levels in this analysis are: a≤0.05, b≤0.02, and c≤0.01, respectively. (B) Western blot analysis of TNF-R1, α-SMA, LCN2, and TIMP-1 in WT and *Atp7b* ^-/-^ mice. Brain protein extracts were prepared as outlined in Methods. The expression of β-actin served as a control for equal loading. (PPT 126 KB)

Additional file 2: Figure S2: Reproducibility of LA-ICP-MS measurements in brain tissue. 30-μm thick brain cryo-cuts were prepared from several of the same animals depicted in Figures 2 and 4. The specimens were subjected to LA-ICP-MS measurements using the same experimental setup. Please note the different concentration bars that are ranging from 0-1% for carbon, 0 - 2000 μg g^-1^ for sodium, 0 - 3000 μg g^-1^ for phosphate, 0 - 20 μg g^-1^ for Cu, 0 - 50 μg g^-1^ for Fe, 0 - 50 μg g^-1^ for Zn, 0- 0.2 μg g^-1^ for lead, and 0- 0.5 μg g^-1^ for Mn, respectively. (PPT 5 MB)

Additional file 3: Figure S3: Confirmatory analysis. To avoid errors occurring from animal-to-animal variations, we confirmed our data in additional 3 WD and 3 WT controls. Animals that were subjected to this analysis were aged 19-24 months and images were visually analyzed but not submitted to region of interest based analysis. Representative images of carbon, Cu, Fe and Zn are depicted. (PPT 7 MB)

Additional file 4: Table S1: Primers used in this study. (DOC 30 KB)

Additional file 5: Table S2: Antibodies used in this study. (DOC 42 KB)

## Abbreviations

α-SMA: α-smooth muscle actin
*Atp7b*: Gene encoding the ATPase-dependent Cu^2+^−transporting beta polypeptide
LA-ICP-MS: Laser ablation inductively coupled plasma mass spectrometry
LCN2: Lipocalin 2
MMP-9: Metalloproteinase-9
PVR: Periventricular regions
TIMP-1: Tissue inhibitor of metalloproteinase-1
WT: Wild type.
