# Supplementary material for: Simultaneous monitoring of cerebral metal accumulation in an experimental model of Wilson’s disease by laser ablation inductively coupled plasma mass spectrometry
Source: BMC Neurosci. 2014 Aug 20;15:98. doi: 10.1186/1471-2202-15-98 (PMC4156608; doi:10.1186/1471-2202-15-98)
Supplement: Supplementary file 1 — Additional file 1: Figure S1: Comparative analysis of gene and protein expression in brains of wild type and Atp7b -/- mice. (A) Relative mRNA expression of IL-1β, TNF-α, NLRP3, ASC, TIMP-1 and MMP-9 expression in brain of WT and Atp7b deficient mice. Significance levels in this analysis are: a≤0.05, b≤0.02, and c≤0.01, respectively. (B) Western blot analysis of TNF-R1, α-SMA, LCN2, and TIMP-1 in WT and Atp7b -/- mice. Brain protein extracts were prepared as outlined in Methods. The expression of β-actin served as a control for equal loading. (PPT 126 KB) [file 12868_2014_3790_MOESM1_ESM.ppt]

## Slide 1
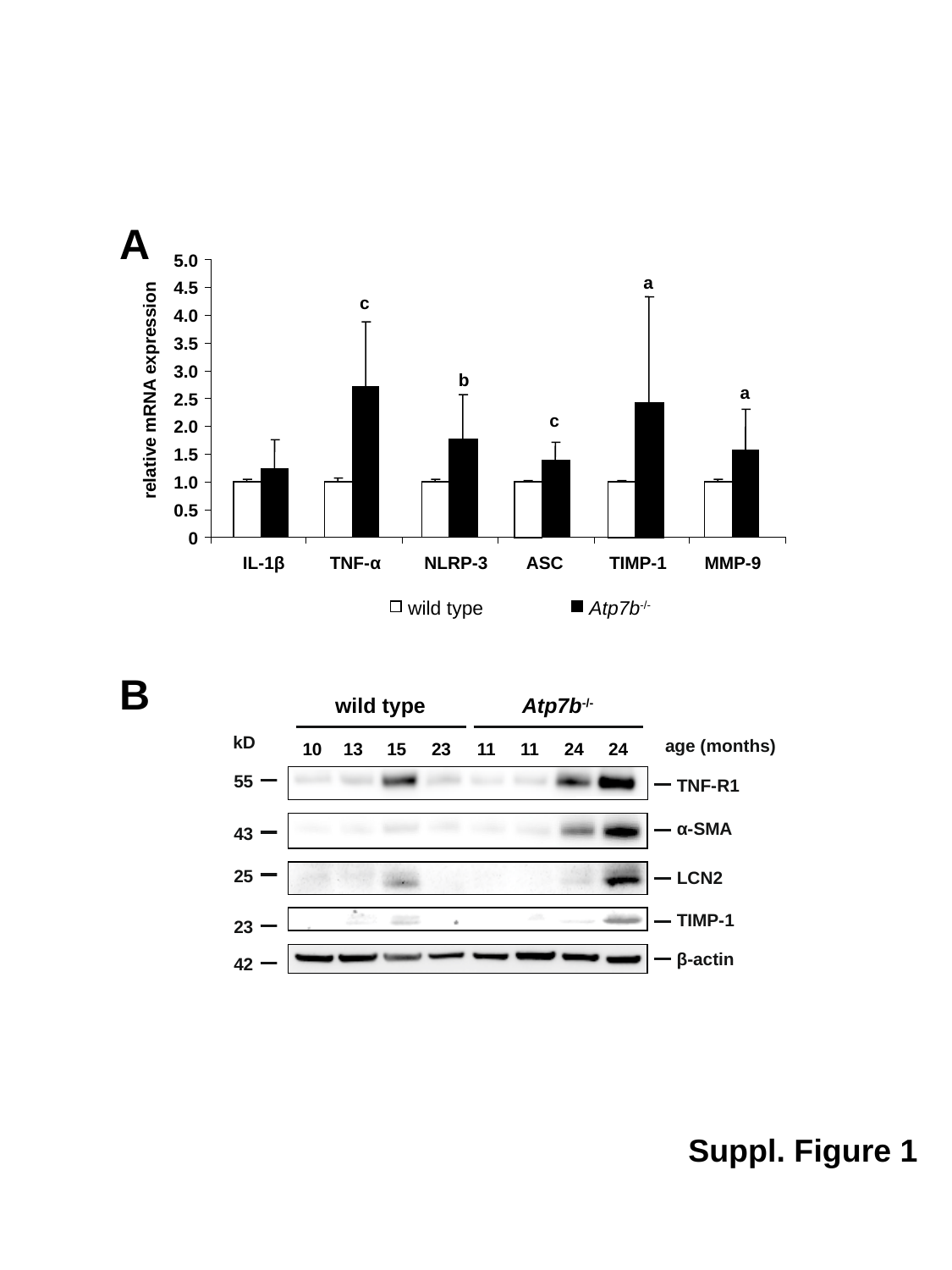

A
5.0
a
TIMP-1
4.5
 c
TNF-α
4.0
3.5
3.0
 b
NLRP-3
 a
MMP-9
relative mRNA expression
2.5
c
ASC
2.0
IL-1β
1.5
1.0
0.5
0
wild type
Atp7b-/-
B
wild type
Atp7b-/-
kD
10
13
15
23
11
11
24
24
age (months)
55
TNF-R1
α-SMA
43
25
LCN2
TIMP-1
23
β-actin
42
Suppl. Figure 1
