# Supplementary figures and images for: Simultaneous monitoring of cerebral metal accumulation in an experimental model of Wilson’s disease by laser ablation inductively coupled plasma mass spectrometry
Source: BMC Neurosci. 2014 Aug 20;15:98. doi: 10.1186/1471-2202-15-98 (PMC4156608; doi:10.1186/1471-2202-15-98)

## Slide 1
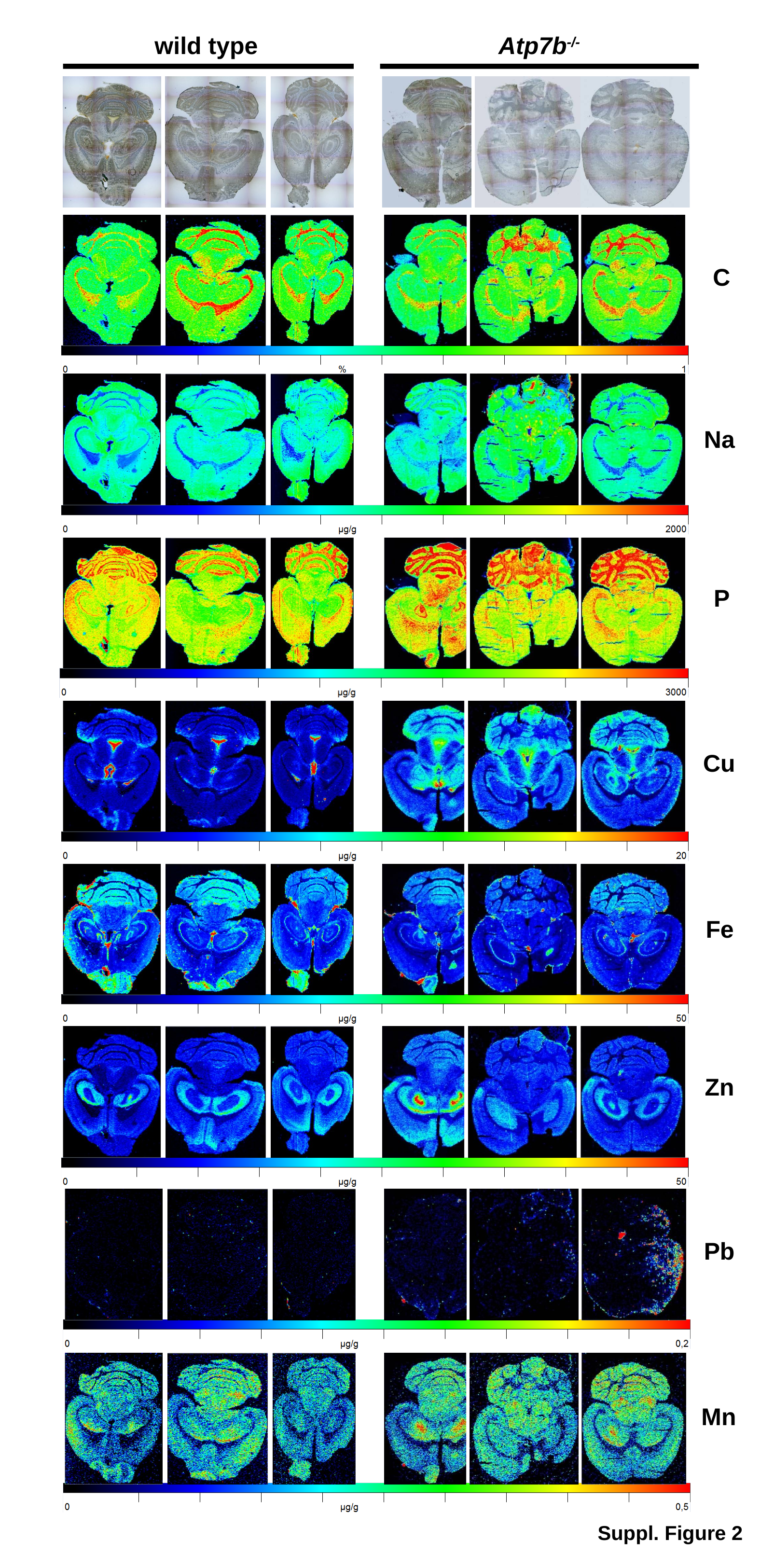

wild type
Atp7b-/-
C
Na
P
Cu
Fe
Zn
Pb
Mn
Suppl. Figure 2

Supplement: Supplementary file 2 — Additional file 2: Figure S2: Reproducibility of LA-ICP-MS measurements in brain tissue. 30-μm thick brain cryo-cuts were prepared from several of the same animals depicted in Figures 2 and 4. The specimens were subjected to LA-ICP-MS measurements using the same experimental setup. Please note the different concentration bars that are ranging from 0-1% for carbon, 0 - 2000 μg g-1 for sodium, 0 - 3000 μg g-1 for phosphate, 0 - 20 μg g-1 for Cu, 0 - 50 μg g-1 for Fe, 0 - 50 μg g-1 for Zn, 0- 0.2 μg g-1 for lead, and 0- 0.5 μg g-1 for Mn, respectively. (PPT 5 MB) [file 12868_2014_3790_MOESM2_ESM.ppt]

## Slide 1
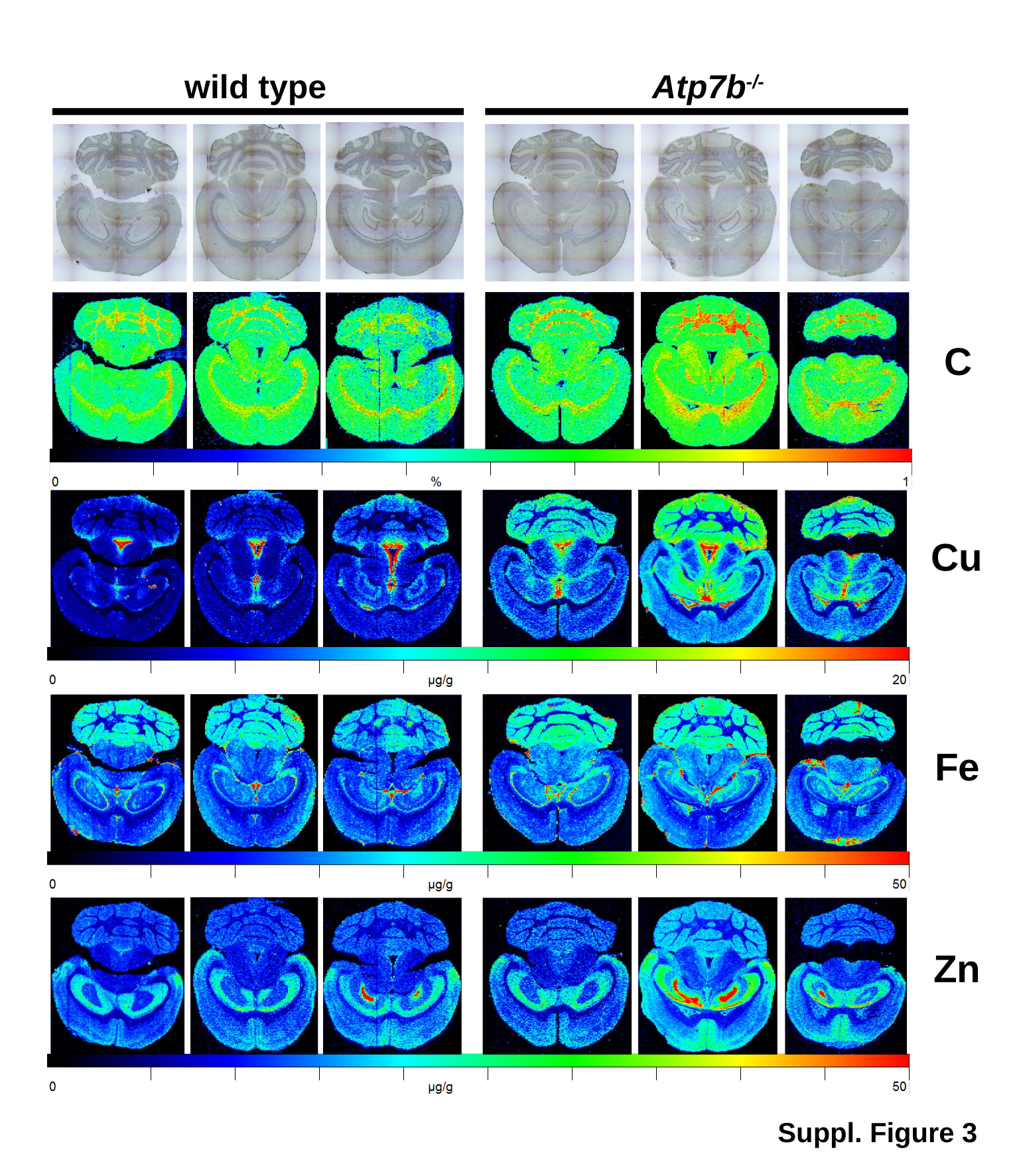

wild type
Atp7b-/-
C
Cu
Fe
Zn
Suppl. Figure 3

Supplement: Supplementary file 3 — Additional file 3: Figure S3: Confirmatory analysis. To avoid errors occurring from animal-to-animal variations, we confirmed our data in additional 3 WD and 3 WT controls. Animals that were subjected to this analysis were aged 19-24 months and images were visually analyzed but not submitted to region of interest based analysis. Representative images of carbon, Cu, Fe and Zn are depicted. (PPT 7 MB) [file 12868_2014_3790_MOESM3_ESM.ppt]
