# Supplementary material for: Simultaneous monitoring of cerebral metal accumulation in an experimental model of Wilson’s disease by laser ablation inductively coupled plasma mass spectrometry
Source: BMC Neurosci. 2014 Aug 20;15:98. doi: 10.1186/1471-2202-15-98 (PMC4156608; doi:10.1186/1471-2202-15-98)
Supplement: Supplementary file 4 — Additional file 4: Table S1: Primers used in this study. (DOC 30 KB) [file 12868_2014_3790_MOESM4_ESM.doc]

**Suppl. Table 1**

Primers used in this study

| **Primers for mouse** | | |
| --- | --- | --- |
| **Gene** | **Acc. No.** | **Primer (5’→ 3’)** |
| mIL-1β | NM_008361.3 | for: TGT AAT GAA AGA CGG CAC ACC  rev: TCT TCT TTG GGT ATT GCT TGG |
| mTNF-α | NM_013693.2 | for: TCT TCT CAT TCC TGC TTG TGG  rev: GGT CTG GGC CAT AGA ACT GA |
| mNLRP-3 | NM_145827.3 | for: CCC TTG GAG ACA CAG GAC TC  rev: GAG GCT GCA GTT GTC TAA TTC C |
| mASC | NM_023258.4 | for: GAG CAG CTG CAA ACG ACT AA  rev: GTC CAC AAA GTG TCC TGT TCT G |
| mTIMP-1 | AY622853 | for: TCC TCT TGT TGC TAT CAC TGA TAG CTT  rev: CGC TGG TAT AAG GTG GTC TCG TT |
| mMMP-9 | NM_013599 | for: CAG GAT AAA CTG TAT GGC TTC TGC  rev: GCC GAG TTG CCC CCA |
| mGAPDH | XM_001473623 | for: ACT GCC ACC CAG AAG ACT G  rev: CAC CAC CCT GTT GCT GTA G |
