# Supplementary material for: Simultaneous monitoring of cerebral metal accumulation in an experimental model of Wilson’s disease by laser ablation inductively coupled plasma mass spectrometry
Source: BMC Neurosci. 2014 Aug 20;15:98. doi: 10.1186/1471-2202-15-98 (PMC4156608; doi:10.1186/1471-2202-15-98)
Supplement: Supplementary file 5 — Additional file 5: Table S2: Antibodies used in this study. (DOC 42 KB) [file 12868_2014_3790_MOESM5_ESM.doc]

**Suppl. Table 2**

Antibodies used in this study

| **Antibody** | **Cat. No.** | **Clonality*** | **Supplier** | **Species*** | **Dilution*** |
| --- | --- | --- | --- | --- | --- |
| **Primary antibodies** | | | | | |
| TNF-R1 | sc-8436 | Mono | Santa Cruz | h, m, r | 1:1000 |
| NLRP-3 | sc-66846 | Poly | Santa Cruz | h, m, r | 1:500 |
| TIMP-1 | sc-5538 | Poly | Santa Cruz | h, m, r | 1:1000 |
| α-SMA | CBL171 | Mono | Cymbus | h, m, r | 1:1000 |
| mIL-1β/IL-1F2 | AF-401-NA | Poly | R&D Systems | m | 1:1000 |
| LCN2/NGAL | AF3508 | Poly | R&D Systems | m,r | 1:1000 |
| β-actin | A5441 | Mono | Sigma-Aldrich | h, m, r | 1:10000 |
| **Secondary antibodies** | | | | | |
| IgG-HRP | sc-2004 | N/A | Santa Cruz | r | WB: 1:5000 |
| IgG-HRP | sc-2005 | N/A | Santa Cruz | m | WB: 1:5000 |
| IgG-HRP | sc-2056 | N/A | Santa Cruz | g | WB: 1:5000 |

* Abbreviations used are: Mono, monoclonal antibody; Poly, polyclonal antibody; N/A, not applicable; h = human, m = mouse, r = rat, g =goat.
